# Parallel imaging: is GRAPPA a useful acquisition tool for MR imaging intended for volumetric brain analysis?

**DOI:** 10.1186/1471-2342-9-15

**Published:** 2009-08-03

**Authors:** Terri L Lindholm, Lisa Botes, Eva-Lena Engman, Anders Frank, Tomas Jonsson, Leif Svensson, Per Julin

**Affiliations:** 1 Department of Diagnostic Medical Physics, Karolinska University Hospital Huddinge, Stockholm, Sweden; 2 SMILE Image Laboratory, Karolinska Institute, Stockholm, Sweden; 3 Astra Zeneca Research and Development, Södertälje, Sweden; 4 Department of Rehabilitation Medicine, Danderyd Hospital, Karolinska Institute, Stockholm, Sweden

## Abstract

**Background:**

The work presented here investigates parallel imaging applied to T1-weighted high resolution imaging for use in longitudinal volumetric clinical studies involving Alzheimer's disease (AD) and Mild Cognitive Impairment (MCI) patients. This was in an effort to shorten acquisition times to minimise the risk of motion artefacts caused by patient discomfort and disorientation. The principle question is, "Can parallel imaging be used to acquire images at 1.5 T of sufficient quality to allow volumetric analysis of patient brains?"

**Methods:**

Optimisation studies were performed on a young healthy volunteer and the selected protocol (including the use of two different parallel imaging acceleration factors) was then tested on a cohort of 15 elderly volunteers including MCI and AD patients. In addition to automatic brain segmentation, hippocampus volumes were manually outlined and measured in all patients. The 15 patients were scanned on a second occasion approximately one week later using the same protocol and evaluated in the same manner to test repeatability of measurement using images acquired with the GRAPPA parallel imaging technique applied to the MPRAGE sequence.

**Results:**

Intraclass correlation tests show that almost perfect agreement between repeated measurements of both segmented brain parenchyma fraction and regional measurement of hippocampi. The protocol is suitable for both global and regional volumetric measurement dementia patients.

**Conclusion:**

In summary, these results indicate that parallel imaging can be used without detrimental effect to brain tissue segmentation and volumetric measurement and should be considered for both clinical and research studies where longitudinal measurements of brain tissue volumes are of interest.

## Background

Age is the strongest predicting factor for dementia. In total, dementia affects more than 25% of those aged over 85 years and between 30 and 50% of those aged over 90 years. Due to the population boom of the 1940's the elderly population is rapidly growing. Thus, the prevalence of dementia is expected to increase significantly over the coming decades. Rapid diagnosis and treatment is therefore of evermore-importance.

One of the most widely accepted imaging biomarkers in the aging process is that of brain tissue atrophy and an increase in cerebral spinal fluid (CSF) volume. Much effort is put into correlating rates of brain atrophy with disease progression [1,2]in order that the imaging may be used as a diagnostic tool [3] rather than depending on mental ability tests only. As yet, there is no cure or clinically available vaccine for Alzheimer's disease (AD) but the rate of disease progression may be reduced if medication is started at an early stage. One target group of patients for disease modifying drugs are those in the early stages of AD [4]. It has been previously reported [5] that a decline in brain parenchyma volume (a rate above that of healthy elderly) is seen before the diagnosis of AD can be made. Thus, it may prove invaluable to identify those persons prior to the onset of clinical AD symptoms.

In the case of longitudinal studies involving dementia patients, small volume changes in the brain parenchyma fraction (BPF) or in regional grey matter (GM) are of significance. Expected BPF changes of less than 1% demands that the repeatability in measurement is excellent. In order to interpret the measured volume changes as significant the error in repeated measurement must be very small indeed. This requires that exceptional image quality be achieved on multiple occasions, which may coincide with large changes in patient cognition. The first challenge is therefore to ensure that the repeated protocol is suitable for patients who may not be entirely cooperative or be unable to follow instructions. Short imaging times would therefore be of particular interest.

After successful acquisition of the 3-dimentional data, volumetric analysis must then be performed, which is an intricate method subject to several sources of error. Brain segmentation methods rely on the ability to classify correctly the contents of a voxel as one of three tissue classes: CSF, GM or white matter (WM). Partial volume effects are likely the most significant source of error in segmentation and can be attributed to several factors. Firstly, low image resolution leads to an inherent partial volume increase and secondly, slight patient motion will increase the partial volume. (Significant patient motion will cause more severe artefacts rendering the images unsuitable for volumetric analysis.) To minimise partial volume effects, the smallest measurable volume, i.e. the voxel, should be as small as possible.

Thus, the imaging protocol requirements for successful longitudinal imaging of dementia patients can be considered as having a high resolution (small voxel) and short acquisition time (AT).

Unfortunately, increasing the resolution requires a longer acquisition time in discord with the first goal of shorter acquisition time. However, parallel imaging techniques such as GRAPPA and SENSE [6-8] are now commonly available on clinical MRI scanners using phased array coils for data acquisition. The techniques under-sample k-space and use sensitivity maps of each coil element to predict the signals in missing k-space lines. Thus an acceleration of the acquisition is performed allowing high resolution acquisitions in shorter periods of time (or higher resolution acquisitions in a given AT).

Of course, there is a cost to using parallel imaging and that is a decreased signal to noise ratio (SNR). Since tissue segmentation methods rely on classifying a voxels signal intensity to one of several classes, it is assumed that a decreased SNR reduces the accuracy of volumetric tissue segmentation and to the authors knowledge has not been applied to any longitudinal volumetric studies. Parallel imaging was not, for example, implemented in the most widely known imaging protocol of the Alzheimer's Disease Neuroimaging Initiative (ADNI) [9,10].

The purpose of this study was to test the roll of the GRAPPA parallel imaging technique applied to T1-weighted high resolution imaging intended for volumetric analysis. A study was recently published looking at the effect of SENSE parallel imaging applied to 3D T1-weighted image acquisitions on Voxel-based Specific Regional Analysis for Alzheimer's Disease (VSRAD). Very little difference was found between the images acquired with and without SENSE applied despite the reduced SNR native to SENSE images. [11] Investigations were performed on both young healthy and elderly volunteers including AD as well as mild cognitively impaired (MCI) patients. Global brain tissue volumes were repeatedly measured in both young healthy volunteers and in the elderly dementia groups.

The noise distribution in an image acquired with parallel imaging is not uniformly distributed but is worse at the centre of the imaging volume. Thus, the ability to quantify volumes in the one of the most compromised regions was also tested by measurement of hippocampus volumes in dementia patients using manual outlining methods.

## Methods

### Magnetic resonance imaging

A 1.5 T Avanto (Siemens Healthcare, Erlangen, Germany) scanner equipped with a 12-channel phased array matrix coil were used in this study. It was decided that a complete 3D imaging protocol was most suitable since it allows high SNR and high resolution in all three directions for each sequence. The 3D data sets were all acquired in the sagital direction. This minimised acquisition time due to the dimensions of the head.

A cubic voxel (1.3 × 1.3 × 1.3 mm) was set since this enables the images to be re-sliced in any of the three major planes without compromising resolution. Volumetric evaluation of small brain structures such as the hippocampus, which is commonly performed in the coronal orientation, could easily be performed after re-slicing the images.

The 3D protocol comprised of Siemens 3D T1-weighted MPRAGE, 3D- FLAIR and 3D T2-weighted SPACE sequences. All three sequences were acquired using the GRAPPA parallel imaging technique [6]. The FLAIR sequence was intended for use in the clinical protocol only and was not used in the brain segmentation process.

The influence of GRAPPA applied to the MPRAGE sequence in particular was investigated since the resulting 3D data are most commonly used for brain tissue segmentation. The MPRAGE sequence was defined with the same imaging parameters as the ADNI MPRAGE sequence. Table 1 includes the imaging parameters of each of the sequences used in this volumetric study. The MPRAGE sequence takes 7 minute 42 seconds for a typical brain acquired in the sagital orientation without GRAPPA. The same sequence acquired with acceleration factor two takes 4 minutes 21 seconds and with acceleration factor four takes 2 minutes 40 seconds. The GRAPPA technique makes use of 24 extra lines of data sampled in the centre of k-space to correct for the missing data throughout k-space thereby maintaining much of the contrast information. Due to the overall under-sampling of k-space, the SNR throughout the image is nevertheless reduced.

**Table 1:** Imaging parameters of volumetric sequences.

Sequence name	MPRAGE	MPRAGE	SPACE
Orientation	Sagittal	Sagittal	Sagittal

TR (ms)	2400	2400	3200

TE (ms)	3.44	3.44	434

TI (ms)	1000	1000	-

Flip angle (degrees)	8	8	Variable T2W

Slice width (mm)	1.3	1.3	1.3

No. Slices per slab	128	128	128

Slice separation (%)	50	50	

TF	-	-	107

Matrix (PE, FE)	192 × 192	192 × 192	192 × 192

FOV (mm)	250	250	250

Phase encoding direction	Anterior-posterior	Anterior-posterior	Anterior-posterior

Bandwidth (Hz/Px)	150	150	704

iPAT	2	4	2

Total scan time (min:s)	4:21	2:40	3:22

The key imaging parameters are shown for the MPRAGE with iPAT acceleration factors 2 and 4 as well as the SPACE sequence.

### Healthy young volunteer

To investigate the repeatability of the scanning-segmentation process a volunteer was repeatedly scanned using the MPRAGE sequence at different parallel imaging acceleration factors. The SPACE sequence was also acquired. The volunteer was scanned 18 times: Six times without parallel imaging, six times with acceleration factor two and six times with acceleration factor four. This scanning was divided into 2 scan sessions within a period of a week. Each scan session was approximately 1 hour in duration and included 3 acquisitions at each parallel imaging factor. Between every scan, the volunteer was removed from the scanner and asked to walk around the room. This ensured that the B1 field inhomogeneity would vary between scans, as would be the case in a longitudinal study.

### Elderly patient group

This clinical study was designed to test the complete protocol on elderly patients whose brains have different anatomy and signal intensity distributions to healthy young volunteers. 15 age-matched elderly patients were recruited through the hospital's Memory Clinic: four patients clinically diagnosed with AD (mean age 70 years; standard deviation 6 years), four MCI patients (mean age 67 years, standard deviation 5 years) and seven healthy elderly patients (mean age 74 years; standard deviation 8 years). The healthy elderly and AD patients were diagnosed using the DSM-IV criteria and MCI patients were defined as subjects investigated for suspected dementia, with cognitive impairment not severe enough to fulfill the criteria for AD.

The 3D protocol (MPRAGE, SPACE and FLAIR sequences) was followed on all 15 patients. The MPRAGE sequences were acquired with acceleration factor two and acceleration factor four in addition to a 3D T2-weighted SPACE sequence. The volumetric acquisition time was approximately 13 minutes in total.

To test the repeatability of the protocol the elderly group was scanned on a second occasion one week after the first using the same imaging protocol. (Two MCI patients were unintentionally not scanned using acceleration factor four on the second scan occasion).

Approval from the hospital's local ethics committee to perform this project was obtained before commencement of any volunteer or patient scanning. Informed consent was obtained from each volunteer and patient prior to entering the study.

### Image processing

Investigating the use of parallel imaging for use in longitudinal volumetric studies must take account of the post processing stages and not simply the acquisition. Thus, two evaluation techniques were used for automatic segmentation of the datasets to verify that the study is not dependant on the post processing technique. No two evaluation software will produce the same segmented tissue volumes [12] and so comparisons of the resulting brain tissue volumes should not be made. However, the repeatability in measurement for each method is the figure of significance.

In-house software, BMAP/Volstat (Stockholm, Sweden) developed for volumetric brain analysis was used in addition to the FSL software package (Oxford, UK) for whole brain volume measurement. For summary description of the two methods see table 2.

**Table 2:** Summary of the image processing procedures using BMAP/Volstat and BET/Sienax.

	BMAP/Volstat (In-house)	BET/Sienax (FSL)
Original images	T1 MPRAGE	T1 MPRAGE and T2 SPACE

Process	Registration to reference brain (9 parameter)	Registration to reference brain (12 parameter)

Output	T1 in standard space	T1 and T2 in standard space

Process	Brain masking using BMAP (cortical CSF (ICV) contour based on T1) (stepwise region growing algorithm)	Brain masking using BET (cortical CSF (ICV) contour based on T2) (brain contour fitting algorithm)

Output	T1 brain (ICV)	T1 brain (ICV)

Process	Tissue segmentation (Volstat – Fuzzy c-means cluster analysis)	Tissue segmentation (Sienax- Random Markov Fields)

Final output	Grey-, white- and CSF-cluster images	Grey-, white- and CSF-cluster images

The key image processing steps are outlined for registration to standard space, masking of non intra-cranial volume (ICV) tissue and tissue segmentation.

FSL [13] is a standard evaluation tool widely accepted for use in volumetric brain tissue analysis. Image data are co-registered to the FSL template brain, inhomogeneity corrected and masked using the BET algorithm [14] (FSL, Oxford, UK) to extract the brain. The data were finally processed through the Sienax [15,16] algorithm (FSL, Oxford, UK) to segment the data into three tissue classes: CSF, GM and WM. The crisp volumes of these tissue classes were output for analysis. In a crisp segmentation each voxel is characterized by a single tissue type only.

There was some difficulty in masking the images in BET. There was often a significant amount of leakage to tissue outside the brain area as is recently reported [17]. The masking was particularly poor around the orbital region and base of the brain. However, this was solved with the use of the co-registered T2- weighted 3D SPACE data, which was masked without problem. The cortical GM – CSF signal intensity difference is greater on T2-weighted imaging, which is the likely cause of the more successful masking. The resulting mask could be applied to the T1-weighted images providing data of an extracted T1-weighted brain for use in volumetric segmentation.

The in-house software is written on the HERMES (Hermes Medical Solutions, Stockholm, Sweden) platform and used to co-register [18], inhomogeneity correct, mask and segment the 3D datasets. The program BMAP masks with a different aim than the BET algorithm so that cortical CSF is also included in the segmented volumes whereas the BET program masks to the brain grey matter surface and therefore only includes an internal CSF volume. The in-house segmentation program called Volstat is based on a fuzzy c means clustering (FCM) algorithm [19-21]. The algorithm is enhanced by use of the distribution of signal intensity specific to each tissue type. This is used as a weighting factor in the iteration process to decide the tissue composition of a voxel. Fuzzy volume maps as well as crisp volume maps of each of the three tissue classes are output from Volstat for further analysis. Volumes quoted in this study are the fuzzy volumes and can therefore not be compared to the more course crisp volumes output from Sienax.

The HERMES MultiModality program was also used for hippocampus volume measurement using a manual outlining technique. Analysis was performed using only images acquired with acceleration factor two. A single trained operator performed all measurements. The borders of both left and right hippocampi were delineated on each image slice from posterior to anterior to generate a series of regions of interest (ROIs). The first ROI was drawn on the slice where crus of the fornix could be seen in full profile. The measurements ended where the hippocampus disappeared under the amygdala. The summed volume of each ROI gave the hippocampus volume.

The complete evaluation of whole brain segmentation and hippocampus volume measurement was performed on data of each patient acquired on two separate occasions.

### Statistics

Analysis was performed with the Statistica statistical software package (StatSoft Inc., Tulsa, OK, USA). The class 1 intraclass correlation test (ICC) [22,23] was used which is typical test to measure the agreement between two data sets with 1.0 indicating perfect agreement. According to Landis and Koch [24] values greater than 0.81 indicate almost perfect agreement.

## Results

### Healthy young volunteer

**Figure 1:**
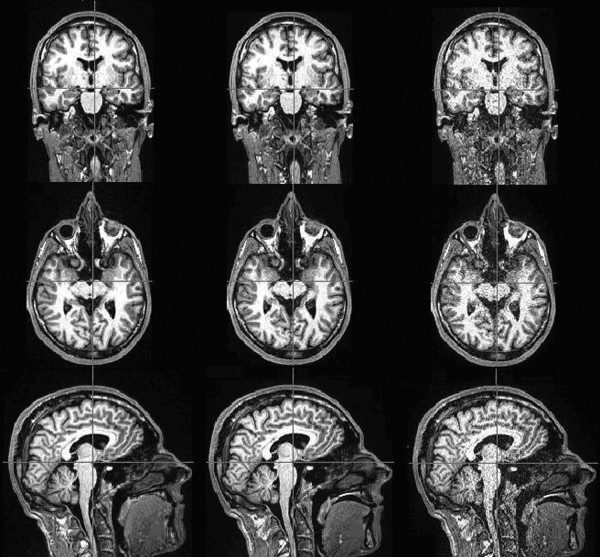
**MPRAGE images acquired without parallel imaging**. Images are acquired with acceleration factor two and acceleration factor four (columns left to right) in each of three perpendicular planes. The sagital direction was that acquired, which was reformatted to the coronal orientation for evaluation of hippocampus measurements and axial orientation for completion.

**Figure 2:**
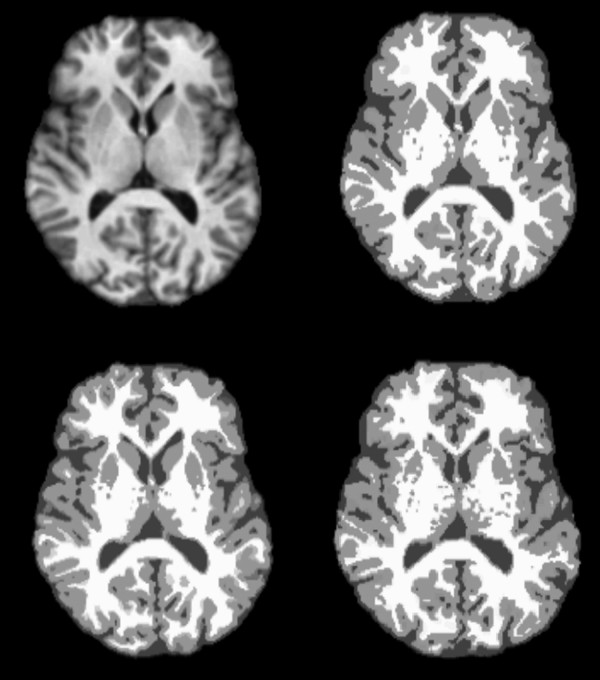
**A transaxial slice of the brain**. The top left image is of a single transaxial slice acquired with an MPRAGE without parallel imaging. The top right image is the same slice post segmentation and crisp volumes of CSF, grey matter and white matter are represented by dark gray, light grey and white regions. The bottom row shows the same slice crisp volumes resulting fro segmentation of MPRAGE images acquired using iPAT acceleration factors 2 and 4 (left and right respectively).

Figure 1 presents a series of images of a single volunteer acquired without parallel imaging and with acceleration factor two and four. It can clearly be seen that the SNR in the image decreases with increasing acceleration factor. However, the SNR loss does not radically affect tissue segmentation as can be seen in figure 2. Even the difficult regions to depict of the basal ganglia are successfully segmented after image acquisition using acceleration factor four.

GM, WM and CSF volumes were measured using both evaluation software packages. The brain parenchyma fraction (BPF) was then calculated. The 18 datasets were grouped according to acceleration factor (without, factor two and factor four) and the mean tissue volume, standard deviation (SD) and coefficient of variation (CoV) were calculated for each group. The descriptive statistics of the repeated scanning of the young healthy volunteer are presented in table 3.

**Table 3:** Descriptive statistics of brain tissue volumes of a young healthy volunteer scanned 6 times at several acceleration (iPAT) factors.

		BET/Sienax			BMAP/Volstat		
Volume	iPAT	Mean	SD	CoV	Mean	SD	CoV

BPF	0	82.9%	0.3%	0.3%	80.7%	0.3%	0.3%

	2	82.3%	0.4%	0.5%	80.6%	0.2%	0.3%

	4	82.4%	0.3%	0.4%	78.1%	0.3%	0.3%

GM	0	823	7.6	0.9%	786	4.2	0.5%

	2	817	6.5	0.8%	786	2.8	0.4%

	4	756	4.0	0.5%	781	2.8	0.4%

WM	0	539	8.5	1.6%	483	3.2	0.7%

	2	553	5.0	0.9%	485	3.8	0.8%

	4	635	3.3	0.5%	452	6.1	1.3%

CSF	0	281	7.3	2.6%	304	4.4	1.4%

	2	294	9.5	3.2%	305	3.3	1.1%

	4	298	7.1	2.4%	345	4.2	1.2%

Volumes were measured using the BET and Sienax algorithms of FSL in addition to the BMAP/Volstat in-house software. Volumes are in cm^3 ^with the exception of BPF, which is unitless.

It is seen that tissue volumes measured with acceleration factor two are remarkably similar to those measured without the application of parallel imaging. However, a more significant change in absolute tissue volumes is observed with the use of acceleration factor four. This is true for brain volumes measured using both evaluation software packages. However, the differences seen using BMAP are considerably smaller than those seen when using Sienax. Moreover, BMAP volumes acquired using acceleration factor two are within 1 SD of the volumes measured without GRAPPA applied whereas volumes measured using Sienax are within 2 SD. Greater deviations in absolute volumes are observed when using images acquired with acceleration factor four with the exception of GM evaluated using BMAP which is within 1 SD. These differences may be due to a difference in tissue classification as a result of the reduced SNR and/or contrast with increasing acceleration. BMAP appears to be less sensitive to such changes, which is likely due to the calculation of fuzzy volumes as opposed to crisp volumes.

Despite the systematic difference in measured volumes using parallel imaging, the tissue volumes are all measured consistently with small coefficients of variation which does not increase with increasing acceleration factor. This is a critical property for use in longitudinal volumetric studies. Analysis using the BET/Sienax program results in a CoV based on the six repeated scans of 0.5% at most for BPF, 3.2% for CSF volume and in the order of 1% for GM and WM volumes. Surprisingly, even the use of acceleration factor four produces tissue volumes with as repeatable measurement as that without the use of parallel imaging.

Analysis using BMAP/Volstat results in even smaller CoV in measurement based on the six repeated scans: 0.3% for BPF, approximately 0.5% for GM and in the order of 1% for CSF and WM volumes. Such precision in measurement indicates that volumes changes over time will be detectable.

### Elderly patient group

All data acquired with parallel imaging acceleration factors two and four were free from motion artefacts and could be used for volumetric analysis. The total and segmented brain tissue volumes were measured using the two evaluation programs for each of the volunteers. The 3D T2-weighted images were successfully used for brain masking in BET. Of the 15 elderly patients who underwent repeated scanning on a second occasion, two MCI patients were unintentionally not scanned using acceleration factor four.

Tissue volumes for all 15 patients were measured and the mean, standard deviation and coefficient of variation were calculated for the entire patient group and presented in table 4. This was performed separately for each acceleration factor on the two separately acquired datasets allowing comparisons to be made between repeated scans of the same elderly patient and different acceleration factors.

**Table 4:** Descriptive statistics of repeatedly scanned patient brain volumes.

			BET/Sienax				BMAP/Volstat			
iPAT	scan		BPF	GM	WM	CSF	BPF	GM	WM	CSF

2	1	Mean	72.9%	662	504	432	73.5%	657	417	386

2	2	Mean	73.2%	664	507	427	73.6%	656	419	385

4	1	Mean	73.2%	596	564	425	75.5%	670	448	361

4	2	Mean	73.0%	596	562	428	75.4%	670	444	363

2	1	SD	3.8%	77	61	57	2.7%	71	53	45

2	2	SD	4.2%	78	54	69	3.0%	71	50	55

4	1	SD	4.1%	62	62	72	2.8%	60	50	55

4	2	SD	3.9%	61	57	70	2.9%	59	51	56

2	1	CoV	0.5%	11.6%	12.0%	13.2%	0.4%	10.6%	11.2%	15.1%

2	2	CoV	0.6%	11.7%	10.8%	16.1%	0.4%	10.5%	11.7%	12.5%

4	1	CoV	0.6%	10.4%	10.9%	16.9%	0.4%	9.2%	11.9%	14.3%

4	2	CoV	0.5%	10.2%	10.2%	16.3%	0.4%	9.0%	12.1%	14.6%

Data of 15 patient images are included in the acceleration factor two comparison and of 13 patients in the acceleration factor four comparison. Volumes were measured using the BET and Sienax algorithms of FSL in addition to the BMAP/Volstat in-house software. Volumes are measured in cm^3 ^with the exception of BPF, which is unitless.

In agreement with the results from the young healthy volunteer, the standard deviation and CoV do not increase with increasing acceleration factor. There is however, as seen in the young healthy volunteer, a systematic difference in absolute volumes.

An intraclass correlation test (ICC) was used to further analyse these results and are presented in table 5. ICC coefficients of greater than 0.95 resulted for both BPF and segmented tissue volumes independent of the volumetric software package used. These results indicate that volumes can be considered almost identical. This is true for data sets acquired with parallel imaging acceleration factor two and four.

**Table 5:** Intraclass correlation (class 1) results of BPF and segmented tissue volumes.

iPAT	Tissue	ICC (Sienax)	ICC (Volstat)
2	BPF	0.955	0.971

2	GM	0.997	0.994

2	WM	0.958	0.961

2	CSF	0.967	0.975

4	BPF	0.993	0.988

4	GM	0.976	0.996

4	WM	0.962	0.983

4	CSF	0.992	0.994

The brain volumes were measured using patient images acquired on two separate occasions with the same acceleration factor. Data of 15 patient images are included in the acceleration factor two comparison and of 13 patients in the acceleration factor four comparison. Volumes were measured using the BET-Sienax algorithms of FSL (ICC(Sienax)) and BMAP-Volstat program (ICC(Volstat)). Volumes are measured in cm^3 ^with the exception of BPF, which is unitless.

A comparison of the volumes measured at the two different acceleration factors could also be made for 13 patients. Data was grouped according to acceleration factor and compared using the ICC. Results presented in table 6 indicate an almost perfect agreement between the volumes measured with ICC coefficients over 0.82 for all tissue volumes and BPF when measured using BMAP/Volstat. Results for BPF and CSF measured using BET/Sienax were particularly excellent with ICC coefficients greater than 0.97. However, such perfect agreement was not measured for all tissue classes. The GM and WM ICC coefficients were 0.737 and 0.543 respectively indicating only reasonable agreement.

**Table 6:** Intraclass correlation (class 1) results comparing 13 patient brain tissue volumes.

Tissue	ICC (Sienax)	ICC (Volstat)
BPF	0.970	0.827

GM	0.737	0.985

WM	0.543	0.878

CSF	0.977	0.882

The volumes compared were measured from images acquired at acceleration factors two and four using the two analysis programs investigated.

Hippocampus volumes were also measured in all 15 elderly patients. Measurements were made by manually outlining the region on the images acquired with acceleration factor two. The same operator performed all measurements.

Repeated measurements of both left and right hippocampi volumes on the same data resulted in an ICC of 0.965 indicating almost identical repeated measurements. This shows that the operator error is exceeding low. Volumes measured on data acquired on two separate occasions were also compared. An ICC of 0.884 resulted, indicating almost perfect agreement in the measurements.

## Discussion

The aim of this study was to investigate if parallel imaging may be a useful technique to shorten image acquisition time in high-resolution volumetric investigations of dementia patients. The authors are not aware of parallel imaging previously being tested for use in volumetric MRI studies. This is particularly important since elderly patients often have difficulties to keep still long enough to acquire such high-resolution images often resulting in poor image quality acquisitions. Today this is solved by repeating the acquisition and hoping for better cooperation. In fact, in the ADNI protocol the MPRAGE sequence is acquired twice in the hope that at least one image is free from motion artefacts.

A group of 15 elderly patients including healthy controls, those diagnosed with mild cognitive impairment and Alzheimer's disease underwent MRI examinations to acquire high-resolution images using the GRAPPA parallel imaging technique. All patients were successfully examined and resulting images were artefact free. Image acquisition times were shortened from approximately 7.5 minutes to 3.5 minutes.

The increased number of clinical trials regarding symptomatic and disease modifying drugs highlights a need to monitor not only healthy elderly controls but also more severely affected AD patients. Such patients are often the most uncooperative and are unable to remain still for the prolonged periods required for an MRI examination. Typical existing imaging protocols often include high-resolution 3D images that are time consuming to acquire (8–10 mins). These long scan times lead to a higher risk of such patients moving during acquisition causing motion artefacts that prevent volumetric analysis from being performed. This leads to a rejection or dropout of patients who are severely affected by neurodegenerative diseases and thereby leading to a possible biasing of clinical data towards patients in the earlier stages of the disease. Thus, the need for short acquisition times is paramount to ensuring comprehensive volumetric data can be collected that is representative of the complete spectrum of dementia patients. The protocol presented here with the application of GRAPPA acceleration factor 2 would reduce the scan time in half which would likely result in a reduced drop out rate of patients in AD studies and clinical drug trials.

The results presented here indicate that parallel imaging can indeed be used for high-resolution (1.3 × 1.3 × 1.3 mm) image acquisition at 1.5 T without hampering automatic or manual volumetric segmentation methods. It is apparent from the descriptive statistics presented in table 4 that the imaging protocol with parallel imaging can be reliably used to measure brain-tissue volumes in the elderly patient group. Two volumetric analysis software programs have been used to evaluate all data. Repeated measurements of tissue volumes made using images acquired on two separate occasions are almost identical with ICC coefficients of greater than 0.95 (table 5) for all tissue classes independent of the software used for volumetric analysis.

The BPF appears unchanged with the application of GRAPPA acceleration factor two compared to the use of no parallel imaging (tables 3 and 4). This is true independent of the software choice for volumetric analysis. In fact, the BPF appears essentially unchanged even with the application of acceleration factor four when segmentation was performed using Sienax (tables 3 and 4). ICC tests comparing BPF measured with parallel imaging factors two and four evaluated with Sienax result in a coefficient of 0.970 (table 6). The coefficient of variation in repeated measurement of the BPF in the same volunteer was no greater than 0.5% for all acceleration factors investigated and is independent of the software used.

However, absolute tissue volumes appear to change with parallel imaging acceleration factor as presented in tables 3 and 4 and compared in table 6. This is attributed to the increase in image noise, which affects the SNR and image contrast and thereby the tissue classification algorithms. The Sienax algorithm appears to consistently measure CSF volume as the acceleration factor is increased but the distribution of grey and white matter changes. Thus, the BPF remains constant. Whereas, the Volstat software appears to consistently measure GM volume but that of WM and CSF changes marginally when using acceleration factor four, thereby affecting the BPF. Further study is necessary to investigate the mechanism of this effect paying particular attention to the non-uniform noise distribution throughout the images.

There is also, as expected, some difference observed in the absolute brain tissue volumes measured when images were analysed with different software (tables 3, 4). The BMAP software includes cortical CSF whereas the BET software includes only internal CSF. Thus, the BPF measured with Volstat is smaller than that measured using Sienax. The method of tissue classification is also significantly different and accounts for the differences measured in tissue volumes. Namely, Sienax measured crisp volumes whilst Volstat measured fuzzy volumes after weighting the signal intensity distribution according to the spread in signal intensity peaks. Previously published work [12] has shown that different volumetric software used to evaluate the same images result in different absolute tissue volumes. The figure of merit however, when comparing volumetric tools, is that of reproducibility in measurement. Both software tested in this study handled the parallel imaging data successfully with high repeatability in measurement (table 5), although T2-weighted images were required to successfully mask the brain when using the BET algorithm of FSL. Since these images are acquired as part of the routine examination this is not considered a problem.

Images acquired using GRAPPA parallel imaging factor two were used to manually outline the hippocampus of all elderly patients. Hippocampi volumes were measured using two images of the same patient and ICC tests indicate almost perfect agreement in measurements. This indicates that despite the reduced SNR in the centre of the image, parallel imaging with acceleration factor 2 does not hinder the manual measurement of hippocampus volumes in elderly patients in longitudinal studies which is also of importance in the study of Alzheimer's disease progression.

Further studies are planned to examine the elderly patient group again which will be more approximately two years after the initial examinations. The effect the parallel imaging factor has on the measured BPF change will be investigated.

## Conclusion

In summary, these results indicate that at 1.5 T and a resolution of 1.3 × 1.3 × 1.3 mm parallel imaging can be used without detrimental effect to brain tissue segmentation and volumetric measurement of global and regional tissue volumes. The acquisition time for the necessary high-resolution T1-weighted scan can be reduced by almost a factor of two. In this study, all patients were examined without the need to repeat any acquisition due to patient movement. Those designing the imaging protocol for longitudinal volumetric studies should consider the application of parallel imaging from the outset. This may allow easier recruitment of many patients into the study and minimise the drop-out rate over the duration of the study as patient health deteriorates with age.

## Competing interests

The authors declare that they have no competing interests.

## Authors' contributions

TL participated in study design and optimisation of imaging method, performed statistical analysis, participated in the interpretation of data and prepared the manuscript.

LB measured total and regional brain tissue volumes in the patient group.

ELE participated in development of software for automatic brain tissue segmentation and tissue volume measurement.

AF co-ordinated the project and participated in scientific design of study.

TJ participated in study design and optimisation of imaging method.

LS participated in development of software for automatic brain tissue segmentation and tissue volume measurement, participated in the interpretation of data and preparation of manuscript.

PJ participated in development of software for automatic brain tissue segmentation and tissue volume measurement and participated in the interpretation of data.

All authors have read and approved the final manuscript.

**Presentation in part at meetings**: TL Watson, T Jonsson, L Botes et al. Magnetic Resonance Parallel Imaging in the Evaluation of Brain Volumes. International Conference on Alzheimer's Disease (ICAD): Alzheimer Imaging Consortium 2006.

## Pre-publication history

The pre-publication history for this paper can be accessed here:

http://www.biomedcentral.com/1471-2342/9/15/prepub
